# The use of private regulatory measures to create healthy food retail environments: a scoping review

**DOI:** 10.1017/S136898002400065X

**Published:** 2024-03-11

**Authors:** Jane Dancey, Belinda Reeve, Alexandra Jones, Megan Ferguson, Emma van Burgel, Julie Brimblecombe

**Affiliations:** 1 Department of Nutrition, Dietetics and Food, Monash University, Clayton, VIC, Australia; 2 The University of Sydney Law School, Sydney, NSW, Australia; 3 The George Institute for Global Health, University of New South Wales, UNSW, Sydney, NSW, Australia; 4 School of Public Health, The University of Queensland, Herston, QLD, Australia

**Keywords:** Food environment, Healthy food retail, Regulation, Governance, Contract

## Abstract

**Objective:**

Different forms of public and private regulation have been used to improve the healthiness of food retail environments. The aim of this scoping review was to systematically examine the types of private regulatory measures used to create healthy food retail environments, the reporting of the processes of implementation, monitoring, review and enforcement and the barriers to and enablers of these.

**Design:**

Scoping review using the Johanna Briggs Institute guidelines. Ovid MEDLINE, PsycINFO, Embase, CINAHL Plus, Business Source Complete and Scopus databases were searched in October 2020 and again in September 2023 using terms for ‘food retail’, ‘regulation’ and ‘nutrition’. Regulatory measure type was described by domain and mechanism. Deductive thematic analysis was used to identify reported barriers and enablers to effective regulatory governance processes using a public health law framework.

**Setting:**

Food retail.

**Participants:**

Food retail settings using private regulatory measures to create healthier food retail environments.

**Results:**

In total, 17 694 articles were screened and thirty-five included for review from six countries, with all articles published since 2011. Articles reporting on twenty-six unique private regulatory measures cited a mix of voluntary (*n* 16), mandatory (*n* 6) measures, both (*n* 2) or did not disclose (*n* 2). Articles frequently reported on implementation (34/35), with less reporting on the other regulatory governance processes of monitoring (15/35), review (6/35) and enforcement (2/35).

**Conclusions:**

We recommend more attention be paid to reporting on the monitoring, review and enforcement processes used in private regulation to promote further progress in improving the healthiness of food retail environments.

Unhealthy diets and associated adverse health conditions including overweight and obesity are a seemingly intractable global challenge related to contemporary global food systems^(1,2)^. It is estimated that 11 million deaths globally were attributable to dietary risk factors in 2017, with the most important risk factors being high intakes of Na and low intakes of whole grains and fruits^(1)^.

Within its Global Action Plan to prevent and control non-communicable diseases (NCDs), the WHO encourages its Member States to develop and implement a range of measures to promote healthier diets, including actions that address the food environment^(3)^. With respect to the food retail environment, where food is sold to, and purchased by consumers, WHO specifically recommends ‘policy measures that engage food retailers and caterers to improve the availability, affordability and acceptability of healthier food products’^(3)^. Recommended strategies to prevent diet-related conditions increasingly include measures which seek to regulate the food environment to decrease the health and economic burden of NCD^(4–6)^. Public health research describes a range of regulatory interventions that seek to enable healthy food purchases by consumers by targeting the food environment^(4,7,8)^.

Food retail regulatory interventions can take a variety of forms and involve both government and non-government stakeholders. For the purposes of this review, we differentiate between forms of regulation developed by government, also called ‘public regulation’ (e.g. reformulation programmes, front of pack labelling, sugar taxes, zoning/bylaws limiting the opening of new unhealthy food retail outlets)^(7,9–11)^, and forms of ‘private regulation’ developed by non-government actors, such as arrangements between organisations and retailers or food retail organisations themselves (e.g. policies or contracts specifying the type, labelling, amount or placement of healthy food or beverages in food retail and vending)^(12–14)^.

While there has been significant focus in the academic literature and international policy recommendations on public regulation, different forms of private regulation are increasing at both national and global levels, including in the regulation of food retail environments^(13)^. Private or multi-stakeholder forms of regulation are increasingly used to address issues such as fair food trading, food safety and environmental sustainability in food retailing (as with fair trading certification schemes developed by non-government organisations and business actors)^(15–17)^.

Private regulation can be voluntary or mandatory in nature, that is, enforceable. Voluntary private regulation relies on the agreement of the regulated entity (the food retailer in the case of this review) to implement, and there are no enforceable consequences for non-compliance. Enforceable private regulation includes contractual obligations often found in vending contracts to provide a certain percentage of healthier food options, accompanied by mandatory sanctions for non-compliance including dismissal of the vendor^(18)^.

Available evidence suggests challenges in implementing effective private regulation to support healthy food retail environments^(19–22)^. Where they have been attempted, such interventions are often externally driven and maintained by health sector actors, with variable interest from food retailers themselves^(7,23)^. Various barriers (lack of customer demand, lack of retailer interest in menu labelling and lack of standardised recipes) and enablers (improved business image, consumer interest and competitive advantage) have been identified^(24,25)^. However, the provision and promotion of healthy food in food retail settings remains difficult to implement and sustain^(22)^. Existing studies indicate that food retailers can perceive interventions like menu labelling as a potential threat to profit and without specific intervention from the public health community, retailers currently have little incentive to independently label, promote and sell healthier food items^(24,26)^.

Further, research from the fields of regulation and public health law show that in order to be effective, all regulatory measures must be accompanied by adequate processes for monitoring, enforcement and review^(27,28)^. The inclusion of monitoring processes allows for an evaluation of the regulatory measure’s performance in achieving its objectives and enables enforcement action (for mandatory schemes)^(29)^. Likewise, processes of review and enforcement are important for enabling continuous improvement, deterring non-compliance and enhancing the credibility of private regulation. Ideally, monitoring, enforcement and review processes should be undertaken by external, independent actors, although this is relatively rare in private regulatory systems^(29)^. This review, therefore, places a novel focus on the use of private regulation in food retail settings that has the aim of improving diet-related health with a specific focus on the processes used to implement this form of private regulation. Drawing on insights from public health law and regulatory theory, this review examines the types of private regulatory measures used to create healthy food retail environments, how these measures were implemented, monitored, reviewed and enforced, and the barriers to and enablers of these effective regulatory governance processes. In doing so, this review contributes to the emerging area of healthy food retail research in public health nutrition.

## Methods

### Protocol and registration

We undertook a scoping review informed by the Johanna Briggs Institute guidelines for scoping reviews^(30)^ and reported according to the Preferred Reporting Items for Systematic Reviews and Meta-analysis extension for Scoping Reviews (PRISMA-ScR)^(31)^. The review protocol was developed by our team of public health nutrition and public health law researchers prior to registration with Open Science Framework https://osf.io/7th83.

### Definitions and eligibility criteria

For the purposes of this research, we defined ‘food retail’ as any physical location that sells food for consumer consumption where the consumer has a choice in regard to what they will purchase^(32–35)^. We included take-away food outlets, supermarkets, restaurants, cafes, vending machines and hospital cafes and excluded online food environments and institutionalised food service (where food is provided free of charge and with no or limited consumer choice) found in settings such as aged care, defence, hospitals and correctional services settings.

Our definition of private regulation includes regulatory measures developed by private actors to implement guidelines or policies developed by public (government) actors. For example, a national or state government may produce a healthy eating framework that they encourage organisations to implement within their own settings^(36,37)^. Where such frameworks are locally implemented by an organisational policy or contract, we include this as an example of private regulation that falls within the scope of this review.

To describe the types of private regulatory measures, we used a framework developed by Mozaffarian that classifies policy interventions by level, target, domain and mechanism^(38)^. Originally developed to analyse features of government-led (public) regulation, we adapted this tool to suit our focus on private regulation and used it to extract information on the domain and mechanism for each regulatory measure (Table 1). For our purposes ‘domain’ refers to the broad type of action or intervention used and includes instore point of purchase information, fiscal policies (e.g. pricing strategies), food quality standards (percentage of healthy items offered for sale) and built environment changes (e.g. changing the physical environment to favour the selection of healthier foods). ‘Mechanism’ refers to the modification the intervention is attempting to achieve and includes attempts to alter consumer preference or choice, altering the composition of food sold so it is healthier (prepared or pre-packaged products with less salt/sugar/fat), and altering the availability and accessibility of healthier food options in the food retail setting.

**Table 1:** Study Characteristics

Publication details	Country	Initiative name	Aim	Who developed the regulation?	Who does the regulation apply to (as described in the research)?	Population being studied in the research	Domain	Mechanism
An, 2017^(39)^	South Africa	HealthyFood program is part of Discovery insurance company’s, health promotion program ‘Vitality’.	A rebate program to promote healthy diets among privately insured health plan members by providing a cash rebate for healthy food purchases in supermarkets.	Discovery Private Health Insurance	Discovery Insurance Company identified 6000 healthier products in *Pick n Pay* supermarkets which are eligible for the HealthyFood benefit by Discovery customers	432 supermarkets, 330 000 eligible health fund members	Economic incentive	Food accessibility
Anzman-Frasca, 2015a^(40)^	USA	Kids LiveWell standards	National Restaurant Association’s Kids LiveWell (KLW) program aims to make children’s meal orders healthier, with limited change to price and revenue following the implementation of a healthier children’s menu in a full-service restaurant chain.	National Restaurant Association (a trade industry association)	Silver Diner full service restaurant chain	13 Silver Diner restaurants	Food standards; point of purchase information	Altering consumer preference or choice; food formulations; food availability
Anzman-Frasca, 2015b^(41)^	USA	Kids LiveWell standards	National Restaurant Association’s Kids LiveWell (KLW) program aims to make children’s meal orders healthier, with limited change to price, and revenue following the implementation of a healthier children’s menu in a full-service restaurant chain.	National Restaurant Association (a trade industry association)	Silver Diner full service restaurant chain	13 Silver Diner restaurants	Food standards; point of purchase information	Altering consumer preference or choice; food formulations; food availability
Bagwell, 2014^(42)^	England, UK	Healthier Catering Commitment (HCC) Scheme *Note the term ‘catering’ in this paper refers to foods consumed outside the home and provided by food businesses, including fast food outlets.	Healthy Catering Commitment (HCC) is an award scheme to encourage businesses to adopt healthier catering practices.	Developed by the Chartered Institute of Environmental Health (CIEH), in conjunction with a London-wide network of Environmental Health Officers (EHOs)	Small, independent catering businesses in London	First study: 77 businesses across 12 London boroughs. Second study: 10 businesses and 28 customers from five businesses	Food standards; point of purchase information	Food formulation; food availability
Bell, 2013^(43)^	Australia	The Healthier Choices intervention was developed to be supportive of the NSW Health policy directive: Healthier Food and Drink Choices for Staff and Visitors in NSW Health.	An organisational (health district) policy to increase availability of healthier food and drink in food outlets and vending machines in health care facilities, and to ensure they are labelled as such.	New South Wales Health (State Government)	Food retail outlets and vending machine operators in health care facilities in the Hunter New England Region of NSW	Hunter New England Local Health District: 5 food outlets and 90 vending machines	Food Standards; point of purchase information	Food formulation; food availability and accessibility
Blake, 2021^(44)^	Australia	Healthy vending policy as one part of a holistic university food policy (the Deakin Food Charter)	To increase vending ‘green’ and ‘amber’ purchases and decrease ‘red’ purchases.	Deakin University, based on Victorian Government nutrition guidelines	Beverage and snack vending machines	51 beverage vending machines across 4 university campuses in Victoria, Australia	Point of purchase information, food standards	Food availability and accessibility
Boelsen-Robinson, 2019^(12)^	Australia	‘Healthy Choices: Food and Drink Guidelines for Victorian Public Hospitals’ was released by the Victorian State Government and adopted by a large metropolitan health service as a mandated, organisational healthy food policy.	An organisational (health service) healthy food policy aimed at supplying a wider range of healthier foods in food retail outlets in healthcare facilities.	Victorian Department of Health (State Government)	Food and drinks in retail outlets, vending and catering in public healthcare facilities in Victoria	Five retail food outlets (1 excluded) in a health service with 8000–10 000 employees.	Food standards; point of purchase information	Altering consumer preference; food formulations; food availability
Bogart, 2019^(45)^	United States	Better Calories Initiative (BCI)	The Balance Calories Initiative (BCI) is a self-regulatory initiative with two components: (i) a National Initiative to reduce beverage calories; and (ii) a Communities Initiative, which aims to reduce beverage calories in 8–10 US communities with less access to or lower sales of no- or reduced-calorie beverages.	A 2014 partnership between the American Beverage Association (a trade industry association) and the Alliance for a Healthier Generation (a child health NGO)	Food stores and restaurants across the USA	Participants (parents, youth and store/restaurant managers) were drawn from three communities in the USA	Mass media; point of purchase information	Altering consumer preference or choice
Butler, 2011^(46)^	Australia	Mai Wiru (Good Food) Regional Stores Policy	The Mai Wiru (Good Food) Regional Stores Policy aims to remove the three highest selling sugar-sweetened beverages (SSB) from a community store to improve the health and wellbeing of Aboriginal people living on the Anangu Pitjantjatjara Yankunytjatjara (APY) Lands (located 550 km south-west of Alice Springs, with a population of ∼400, and next nearest store ∼100 km away).	APY Lands community members	Remote food store based on APY Lands (only one such store)	Store sales data were examined before and after withdrawal of the three highest selling SSB to determine purchasing patterns, volumes sold, sugar and energy purchased.	Built environment changes	Food availability and accessibility
Choi, 2021^(47)^	United States	Voluntary policy	To create healthier kids’ meals.	Fast food restaurants	Fast food restaurants (McDonald’s, Burger King, Wendy’s, Subway)	Children’s meal purchases from fast food restaurants	Food standards, point of purchase information	Food availability and accessibility, food formulations
Ejlerskov, 2018a^(48)^	England, UK	Checkout food policies	Supermarket-led checkout food policies to reduce unhealthy food product displays at supermarket checkouts.	9 UK supermarkets: Aldi, Asda, Coop, Lidl, M&S, Morrisons, Sainsbury’s, Tesco, and Waitrose	Each organisation’s policy applied to its own supermarkets	9 supermarket groups representing 90 9 supermarket groups representing 90% of the UK grocery market	Built environment changes	Food availability and accessibility
Ejlerskov, 2018b^(49)^	England, UK	Checkout food policies	Supermarket-led checkout food policies to reduce unhealthy food product displays at supermarket checkouts.	9 UK supermarkets: Aldi, Asda, Coop, Lidl, M&S, Morrisons, Sainsbury’s, Tesco, and Waitrose	Each organisation’s policy applied to its own supermarkets	9 supermarket groups representing 90 9 supermarket groups representing 90% of the UK grocery market	Built environment changes	Food availability and accessibility
Ejlerskov, 2018c^(50)^	England, UK	Checkout food policies	Supermarket-led checkout food policies to reduce unhealthy food product displays at supermarket checkouts.	9 UK supermarkets: Aldi, Asda, Coop, Lidl, M&S, Morrisons, Sainsbury’s, Tesco, and Waitrose	Each organisation’s policy applied to its own supermarkets	9 supermarket groups representing 90 9 supermarket groups representing 90% of the UK grocery market	Built environment changes	Food availability and accessibility
Eneli, 2014^(51)^	United States	Institutional policy banning Sugar Sweetened Beverage (SSB) sales	An organisational (hospital) policy to ban SSB in Nationwide Children’s Hospital.	Nationwide Children’s Hospital in Columbus, Ohio	All hospital food establishments (including catering and vending)	Hospital-owned cafeteria, food court, coffee shop, and 2 gift shops; and contracted food service venues (a franchise sandwich shop, an Asian restaurant, and vending machines).	built environment changes	food availability and accessibility
Fandetti,2023^(52)^	United States	Food service contracts	To provide university food services.	The universities sampled	University food service contracts	Food service contracts were collected from 14 North Carolina public universities using food service management companies.	Food standards	Food formulations, food availability and accessibility
Ferguson, 2017^(53)^	Australia	Price discount strategy	Four price discount strategies of 10 Four price discount strategies of 10% aiming to influence grocery, fruit, vegetable and diet soft-drink sales in community stores.	Outback Stores (a retail management organisation)	Eighteen out of 21 community stores managed by Outback Stores who agreed to participate	Eighteen community stores in central, western and northern Australia and 54 informants including local store committee members, store managers, staff and customers	Fiscal Policies	Altering consumer preference or choice, food accessibility
Fildes, 2022^(54)^	UK	Healthy checkouts initiative	To reduce unhealthy foods at supermarket checkouts.	Tesco express convenience stores	Tesco express convenience store checkouts	1151 Tesco express convenience stores	Food standards, built environment changes	Food availability and accessibility
Harpainter, 2020^(55)^	United States	Healthy Default Beverage Standards	A Healthy Default Beverage (HDB) strategy to decrease children’s consumption of SSB.	Multiple Quick Service Restaurants (un-named, *n* 70) instituted their own voluntary HDB standards	Each organisation’s standards applied to their own QSR	205 Quick Service Restaurants (QSR) with and without healthier default beverage policies in 11 Local Health Departments	Built environment changes (HDB is a nudge)	food availability or accessibility
Kirk, 2021^(56)^	Canada	Voluntary nutrition guidelines for recreation and sport settings	To improve children’s dietary intakes.	The recreation centres manage contracts with food service or vending providers	Food retail or vending contracts	11 facilities in Alberta; 14 in British Columbia; and 7 in Nova Scotia; 32 interviews with rec staff managers, committee or board members and rec centre volunteers.	Food standards	Food formulations, food availability and accessibility
Lam, 2018^(57)^	England, UK	Checkout food policies	Nine Supermarket groups with their own checkout food policies aiming to reduce unhealthy food product displays at supermarket checkouts.	Each supermarket group developed their own checkout food policy	Each organisation’s policy applied to its own supermarkets	All stores in 9 supermarket groups open for business in June and July 2017 in a city in Eastern England (population ∼ 125 000)	Built environment changes	Food availability and accessibility
Lane, 2019^(13)^	Canada	Healthier Choices in Vending Machines in British Columbia Public Buildings	Healthy Vending Contracts (HVC) aims to increase healthy/decrease unhealthy products in vending machines in publicly funded sport and recreation facilities by including health stipulations in vending machine contracts.	Ministry of Health, Population and Public Health Division, British Columbia (Provincial Government)	Sport and recreation facilities in public buildings in British Columbia	62 beverage and 43 snack vending contracts within 46 facilities	Built environment changes	Food availability and accessibility
Moran, 2016^(58)^	United States	Healthy Hospital Food Initiative	The Healthy Hospital Food Initiative (HFFI) aims to comprehensively improve the hospital food environment by addressing the nutritional quality of food and beverages purchased and served.	The New York City Department of Health and Mental Hygiene (City Government)	Food retail stores and vending in New York City public and private hospitals	28 Hospitals with cafeterias, cafes and vending machines	Food standards	Food availability and accessibility
Mueller, 2017^(59)^	United States	Kids LiveWell voluntary program	The National Restaurant Association’s Kids LiveWell (KLW) Program aims to improve the healthiness of children’s restaurant meals.	National Restaurant Association (a trade industry association)	Silver Diner full service restaurant chain	5971 checks on children’s menu meal orders in a single full service, regional restaurant after healthy meal changes were implemented	Point of purchase information; food standards; built environment changes (menu format changes)	Altering consumer preference; food formulations; food availability and accessibility
Naughton, 2023^(60)^	Australia	Healthy food and drink policy	To improve the healthiness of sport and aquatic centre food environments for customers and staff.	YMCA, a community organisation that manages over seventy sport and aquatic centres, based on Victorian Government nutrition guidelines	Food and beverage products, placement and promotion in sport and aquatic centres	13 community sport and aquatic centres	Point of purchase information, food standards, built environment changes	Food formulations, food availability and accessibility
Olstad, 2011a^(61)^	Canada	Alberta Nutrition Guidelines for Children and Youth	The Alberta Nutrition Guidelines for Children and Youth (ANGCY) are intended to facilitate children’s access to healthy food and beverage choices within schools, child-care facilities, and recreational facilities.	Alberta Government (Provincial Government)	Recreational facility food retail outlets and vending machine operators in Alberta	Telephone survey to Sport and Rec centres (*n* 151)	Point of purchase information; food standards	Food availability and accessibility
Olstad, 2011b^(62)^	Canada	Alberta Nutrition Guidelines for Children and Youth	The Alberta Nutrition Guidelines for Children and Youth (ANGCY) are intended to facilitate children’s access to healthy food and beverage choices within schools, child-care facilities, and recreational facilities.	Alberta Government (Provincial Government)	Recreational facility food retail outlets and vending machine operators in Alberta	A large, new recreational facility with approximately 50 A large, new recreational facility with approximately 50% of facility users under age 18. Food service was provided by a national chain selling discretionary food. A small, local vending machine company sold unhealthy food and beverages.	Point of purchase information; food standards	Food availability and accessibility
Olstad, 2012c^(63)^	Canada	Alberta Nutrition Guidelines for Children and Youth	The Alberta Nutrition Guidelines for Children and Youth (ANGCY) are intended to facilitate children’s access to healthy food and beverage choices within schools, child-care facilities and recreational facilities.	Alberta Government (Provincial Government)	Recreational facility food retail outlets and vending machine operators in Alberta	Three cases purposefully chosen: 1.) An ANGCY full adopter who adopted and implemented the ANGCY within food retail and vending; 2.) a non-adopter who decided not to incorporate ANGCY recommendations into any of its food service; and 3.) a semi-adopter followed ANGCY recommendations in its vending machines or in its concession(s), but not both.	Point of purchase information; food standards	Food availability and accessibility
Olstad, 2012d^(64)^	Canada	Alberta Nutrition Guidelines for Children and Youth	The Alberta Nutrition Guidelines for Children and Youth (ANGCY) are intended to facilitate children’s access to healthy food and beverage choices within schools, child-care facilities, and recreational facilities.	Alberta Government (Provincial Government)	Recreational facility food retail outlets and vending machine operators in Alberta	The study examined factors influencing adoption and implementation of the ANGCY in publicly funded recreational facilities. Seven managers from industry; five from companies that had adopted and implemented the ANGCY (adopters) in recreational facilities and two from companies that had not (non-adopters).	Point of purchase information; food standards; built environment changes	Food formulations; food availability and accessibility
Pharis, 2018^(65)^	United States	Get Healthy Philly	Get Healthy Philly – Healthy snack and beverage vending standards aim to increase healthy snack and beverage options in vending.	Philadelphia Department of Public Health (State Government)	Vending machines on property owned or leased by City of Philadelphia	Approximately 250 vending machines over a 4-year period	Food standards, fiscal policy; point of purchase information	Altering consumer preference or choice; food availability and accessibility
Rickrode-Fernandez, 2021^(66)^	United States	Food and Beverage Choices (FBC) Policy	University nutrition policies are a useful step toward improving the food environment, leading to improved health outcomes for the campus community.	The University (UC Berkeley)	All campus food provision (the policy established nutrition standards for retail foodservice and markets, vending machines, athletic concessions, dining halls and university-sponsored meetings.)	UC Berkeley food retail settings	Food standards	Food formulations, food availability and accessibility
Robinson, 2019 ^(67)^	England, UK	Kcal Labelling Voluntary Pledge	The Public Health Responsibility Deal – kCal labelling pledge aims to provide point of sale kCal labeling for food served in the eating out of home sector.	UK Department of Health (National Government)	104 eligible restaurant and takeaway chains	Of the 104 eligible chains, only 18 chains provided in store kcal labelling and these were examined	Point of purchase information	Altering consumer preferences or choice
Stead, 2020 ^(68)^	Scotland, UK	Scottish Healthcare Retail Standard	The Scottish Healthcare Retail Standard (HRS) aims to facilitate healthier food choices in healthcare setting.	Scottish Government (National Government)	All public hospital food retail outlets	A purposive sample (*n* 13) of NHS Health Scotland food retail outlets designed to achieve heterogeneity in terms of the following variables: • type of management • health board area • hospital location and catchment area • progress towards HRS compliance at baseline A sample of hospital retail outlets (*n* 17) including shops and trolley services were surveyed using a mixed methods design.	Point of purchase information	Altering consumer preference or choice; food availability and accessibility
vonPhilipsborn, 2018 ^(14)^	Germany	Lidl’s ‘Position Paper Healthy Nutrition’	Lidl’s Nutrition Pledge aims to reduce the average sales-weighted content of added sugar and added salt in its own-brand products by 20 Lidl’s Nutrition Pledge aims to reduce the average sales-weighted content of added sugar and added salt in its own-brand products by 20% until 2025. It also aims to reduce the saturated and trans-fatty acid contents of its own-brand products, without specifying targets or timelines.	Lidl (supermarket chain)	Lidl own-brand products and supermarkets	A major European food retailer (Lidl) with a publicly available nutrition strategy	Food standards and population education through promotion and marketing of healthier foods	Food formulations; altering consumer preference or choice and food availability
Walker, 2020 ^(69)^	Australia	Healthier Drinks at Healthcare Facilities Strategy	The Healthier Drinks at Healthcare Facilities strategy aims to improve the range, availability and promotion of healthy bottled and canned beverage options while limiting the availability of less desirable options.	Queensland Government (State Government)	Beverage sales in all food retail outlets and vending machines in Children’s Health Queensland Hospital and Health Service, Lady Cilento Children’s Hospital	Children’s Health Queensland Hospital and Health Service, Lady Cilento Children’s Hospital. Seven retail food outlets and 14 vending machines.	Point of purchase information, food standards	Altering consumer preference or choice; food availability and accessibility
Wickramasekaran, 2018 ^(70)^	United States	100 100% Healthy Vending Machine Nutrition Policy	The County of Los Angeles Healthy Vending Machine Nutrition Policy aims to provide County employees and the public with greater access to healthier vending machine options at its facilities.	County of Los Angeles Board of Supervisors (County Government)	Vending machines in County of Los Angeles facilities	A vending operator’s quarterly revenue, product sales records, and nutritional information data from 359 vending machines in County of Los Angeles facilities in 2013–2015.	Food standards	Food availability and accessibility

To ensure we captured all articles pertaining to our definition of ‘private regulation’, we used broad search terms for regulation in our initial searches and then excluded forms of ‘public regulation’ at the stage of full-text screening. Searches were limited to articles published in English. We aimed to capture articles that described the use of private regulation that had been embedded in the organisation (i.e. was not a research trial) to create healthy food retail environments. We initially included school food settings in our search; however, this proved problematic. The decision to exclude these articles at the stage of full-text screening was made due to the difficulty of interpreting the results of these articles which reported a combination of both user-pays and institutionalised food service provision. Results were not reported separately according to the different means of food service provision, and therefore these articles were deemed to not meet our inclusion criteria. Our inclusion and exclusion criteria are presented in Table 2.

**Table 2:** Final eligibility criteria

Criteria	Inclusion	Exclusion
Publication date	1 January 2000 – 1 September 2023 (Ovid MEDLINE, Scopus, Embase) and 6 September 2023 (PsycINFO, Business Source Complete)	Prior to 1 January 2000
Language	English	All other languages
publication type	Full text of primary research in peer reviewed literature.	Not peer reviewed; grey literature; opinion piece, conference abstract; review.
Form and intent of regulation	The private regulation must have been used or applied in, or targeted at, creating healthier changes in a *food retail* environment (including vending).	The article did not detail a private regulatory measure used to create healthy food retail The article described a form of public regulation. The regulation was targeted at food retail purchases that send no cue to the consumer (e.g. reformulation strategies).
Sustained private regulation	The regulation was embedded in formal organisational documentation (policy, procedure and strategy) indicating organisational acceptance and longer-term sustainability.	An intervention not embedded in formal organisational documentation.
Settings	Food retail where consumers make a choice to purchase food and/or drink.	School food retail*; institutionalised food service such as aged care, defence, hospitals, correctional, where consumers have no choice to purchase.
Outcome of interest	Article focussed on food and/or drink healthiness to achieve a nutrition outcome.	Article focused solely on other outcomes in the absence of nutrition outcomes; e.g. sustainability, alcohol.

* Added to the exclusion criteria at full-text review.

### Search

A detailed search strategy was developed with the aid of a university librarian and the example for Ovid MEDLINE is presented in see online supplementary material, Supplementary Table S1.

### Information sources

Six databases (Ovid MEDLINE, PsycINFO, Embase, CINAHL Plus, Business Source Complete and Scopus) covering the fields of public health, nutrition, business and law were included in our search strategy to maximise our chances of capturing existing literature. Articles identified from searches conducted by JD on 8–9 October 2020 and repeated on 1 and 6 September 2023 by EvB were downloaded from each of six databases to EndNote and screened for duplicates. Covidence was used to identify and exclude further duplicates and to manage the screening, review and extraction process.

### Selection of sources of evidence

JD and MF independently screened an initial 700 (5 %) titles and abstracts using the inclusion and exclusion criteria and variance was forty-six articles (6·6 %). After discussion, refinements to the criteria were made, and an additional 700 (5 %) of articles were independently screened by both reviewers and variance was thirteen articles (1·9 %). Title and abstract screening on the remaining articles was then conducted by JD with reference to MF for clarification, if required.

### Data charting process

Data were extracted from each article by JD in Covidence using templates designed by the research team. Ten percent of articles were cross checked by a second author to ensure consistency. Data extracted in Covidence were then exported into Microsoft Excel (2018), and the key data were transferred to Microsoft Word (2018) (Tables 1 and 3) and edited for clarity.

**Table 3:** Responsibility for regulatory processes and voluntary/mandatory nature

Publication year	Responsibility for Implementation	Responsibility for Monitoring	Responsibility for Enforcement	Responsibility for Review	Voluntary/ Mandatory
An, 2017 ^(39)^	Discovery Health Insurance	Discovery Health Insurance	Not described	Discovery Health Insurance	Voluntary
Anzman-Frasca, 2015a ^(40)^	Silver Diner Full Service Restaurants	Not described	Not described	Not described	Voluntary
Anzman-Frasca, 2015b ^(41)^	Silver Diner Full Service Restaurants	Not described	Not described	National Restaurant Association	Voluntary
Bagwell, 2014 ^(42)^	EHOs, nutritionists and public health professionals	Not described	Not described	Not described	Voluntary
Bell, 2013 ^(43)^	Good for Kids, Good for Life (a large multi-setting, multi-strategy childhood obesity prevention program) worked with health service management to introduce and implement this policy within the Hunter New England Local Health District (HNELHD) as part of wider efforts to provide healthier environments for children and their families.	Audit monitoring and feedback on vending and food retail but unclear who has responsibility (Good for Kids, Good for Life or HNELHD).	Not described	NSW Health. Reference to future revisions of the policy directive.	Mandatory for vending and hospital owned food outlets. Voluntary for privately owned food retail
Blake, 2021 ^(44)^	University, researchers and vending machine supplier (changes to marketing)	Vending machine supplier provides aggregated monthly electronic sales data pre- and post-intervention. Researchers (from Deakin University) responsible for cross-checking nutrient data Random auditing of approximately 5 machines per month to check proportional displays of red, amber, and green beverages; traffic light labels; and other intervention components.	Any deviations from policy targets are flagged with the vending supplier, who fixes issues within a few days.	Not described	Not described
Boelsen-Robinson, 2019 ^(12)^	The hospital health promotion manager	Not described	Not described	Not described	Mandatory for vending. Voluntary for food retail but *expected at a senior management level*.
Bogart, 2019 ^(45)^	Better Calories Initiative (BCI) created by the American Beverage Association and the Alliance for a Healthier Generation	Not described	Not described	Not described	Voluntary
Butler, 2011 ^(46)^	The Mai Wiru Regional Stores Policy was implemented under the auspices of Nganampa Health Council an Aboriginal owned and controlled health organisation	Not described	Not described	Not described	Mandatory
Choi, 2021 ^(47)^	The fast food retailer (McDonald’s, Burger King, Wendy’s, Subway).	Not described	Not described	Not described	Voluntary
Ejlerskov, 2018a ^(48)^	Each supermarket	Not described	Not described	Not described	Voluntary
Ejlerskov, 2018b ^(49)^	Each supermarket	Not described	Not described	Not described	Voluntary
Ejlerskov, 2018c ^(50)^	Each supermarket	Not described. For the purposes of the study, the study team did instore observations	Not described	Not described	Voluntary
Eneli, 2014 ^(51)^	The hospital. Hospital vendors signed a contract that excluded the sale of SSBs	The hospital established metrics to track the outcome of the SSB ban	Not described	Not described	Mandatory
Fandetti, 2023 ^(52)^	Not discussed	The university department that oversees contracted food services monitors the contract term.	Not described	Many university food contracts have an initial term of 5–10 years, with an option for renewal. This acts as a mechanism for review.	Mandatory
Ferguson, 2017 ^(53)^	Outback stores retail management	Not described	Not described	Not described	Voluntary
Fildes, 2022 ^(54)^	Tesco Express convenience stores	Researchers conducted unannounced store audits	Not described	Not described	Not described
Harpainter, 2020 ^(55)^	Each individual QSR	Not described	Not described	Not described	Voluntary
Kirk, 2021 ^(56)^	The recreation centres in each province	Provincial health promotion organizations “could be considered” as monitoring policy however the degree to which these were present varied in each province. Where a contract is in place, monitoring may be done by the contractor (restocking vending).	Not described	Not described	Voluntary
Lam, 2018 ^(57)^	Supermarkets themselves	Not described	Not described	Not described	Voluntary
Lane, 2019 ^(13)^	Not described	Not described	Not described	Not described	Voluntary
Moran, 2016 ^(58)^	The hospital that adopts the voluntary guidelines. The Health Department offered technical assistance to hospitals, which included provision of implementation guides, promotional materials, and assistance from 2 full-time registered dietitians	The Health Department. Implementation of the standards was monitored through ongoing conversations with hospital staff, site visits, and menu analyses by health department dietitians.	Not described	Not described	Voluntary
Mueller, 2017 ^(59)^	Silver Diner Full Service Restaurant	Not described	Not described	Not described	Voluntary
Naughton, 2023 ^(60)^	Policy implementation was supported by a health promotion officer based at YMCA head office, with each centre responsible for implementing the policy into their own food retail outlet.	Annual auditing was performed by each centre, with audits shared within the organisation to highlight achievements and encourage centres to reach policy targets.	Not described	Not described	Voluntary
Olstad, 2011a ^(61)^	The Recreation facility food service outlet	Not described	Not described	Not described	Voluntary
Olstad, 2011b ^(62)^	The Recreation facility food service outlet	Not described	Not described	Not described	Voluntary
Olstad, 2012c ^(63)^	The Recreation facility food service outlet	Not described	Not described	Not described	Voluntary
Olstad, 2012d ^(64)^	The Recreation facility food service outlet	Not described	Not described	Not described	Voluntary
Pharis, 2018 ^(65)^	The vending companies	The vending companies provided monthly sales data to PDPH from January 2010 to June 2013 as part of its contractual requirement	Not described	Not described	Mandatory
Rickrode-Fernandez, 2021 ^(66)^	The policy team (registered dietitian 10–20hr/week and graduate student fellow 3hr/week).	Student resources trained to conduct audits of food and beverage offering. For meetings and events there is no mechanism to monitor that healthy options are offered. *Recommended* data collection at baseline and follow-up food environment data, sales data, and student and/or staff health indicators and behaviours, to evaluate effectiveness	Noted lack of repercussions for noncompliance. *Recommended* promotional incentives for compliant vendors.	The creators of the FBC policy have built-in regular opportunities to review and revise the standards to ensure that the policy remains relevant and up-to-date with new dietary guidelines and inclusive of new types of food and beverage vendors that are added to the campus food environment.	Voluntary
Robinson, 2019 ^(67)^	Food retail outlets	Not described	Not described	Not described	Voluntary
Stead, 2020 ^(68)^	All food retail outlets, including outlets operated by major national retail groups had an 18-month implementation period concluding in a compliance inspection	A monitoring scheme is run by Scottish Grocers’ Federation (SGF), the trade association for the retail convenience sector in Scotland. SGF provides guidance to retailers on how to meet the HRS requirements and conducts inspections to assess initial compliance. Quality assurance inspections then conducted at least every 2 years	Hospitals have contracts with retail outlets, and adherence to HRS was made a condition of contract renewal; this provided a mechanism for enforcement	The Scottish Government can amend the HRS, indicating it can review. Examples cited are: 1. Promotions: Originally all price-marked packs (packs with the price printed prominently on the packaging) were defined as promotions and therefore not permitted for products not meeting specified nutrition criteria. The HRS rules were amended following feedback from retailers that some items were only available in such packaging. After considering different product sizes, the Scottish Government agreed to allow price-marked packs if the price-marking covered less than 25 % of the pack face 2. Meal deals: Originally only fruit was allowed as the snack item in a meal deal. However, a subsequent increase observed in sales of crisps (and decline in sales of healthier alternatives) led to amendment of the meal deal rules to permit the inclusion of baked crisps	Mandatory
vonPhilipsborn, 2018 ^(14)^	Lidl	Researchers note: the absence of independent monitoring and evaluation	Not described	Researchers noted the absence of independent monitoring and evaluation	Voluntary
Walker, 2020 ^(69)^	A steering committee with hospital, food retail, researchers and consumers. Managers of retail food outlets and vendors were supported by an implementation guide specific to their store or stock. This guide was developed after the baseline audit and detailed the required changes and how they could be achieved, including product suggestions and visual representations of a compliant layout	Audits conducted at baseline, 1-month post implementation and in May 2018 (full implementation occurred in May 2017).	Not described	Not described	Voluntary
Wickramasekaran, 2018 ^(70)^	The County of Los Angeles (County) Board of Supervisors adopted the County of Los Angeles Healthy Vending Machine Nutrition Policy.	The researchers	Not described	Not described	Mandatory

NVivo (2020) software was used to support our qualitative analysis of reported barriers and enablers. JD developed codes in NVivo for barriers to and enablers of implementation, monitoring, review and enforcement of the regulation described and analysed each article. JD and JB compared analyses for 10 % of articles to ensure consistency. A case classification summary report containing all the identified barriers to and enablers of implementation, monitoring, enforcement and review for all thirty-five articles was exported from NVivo to Microsoft Word. Evaluation of the reported barriers to and enablers of regulatory governance processes drew on a framework developed by Reeve and further adapted by other scholars for evaluating and strengthening the performance of public health law and regulation^(28,29,71)^. This framework evaluates the dimensions of regulatory content and the processes established by regulation, including administration/implementation, monitoring, enforcement and review.

### Data items

Data extraction templates were designed to collect data on article demographics, type of regulation including domain and mechanism, voluntary or mandatory nature of the regulation; regulatory governance processes regarding implementation, monitoring, enforcement and review and who had responsibility for them and the barriers to and enablers of these regulatory processes as described by the authors^(38)^. Voluntary regulations were defined by an acceptance from the organisation, institution or food retailer to implement, but with no enforceable consequences for not implementing the regulation. Mandatory regulations defined by an expectation of implementation whether legally binding or organisationally endorsed.

### Synthesis of results

Article demographics, type of regulation, including domain and mechanism, and compulsory nature were descriptively analysed (Tables 1 and 3). Deductive analysis using Reeve and Magnusson’s framework was used to identify barriers and enablers related to monitoring, enforcement and review (Table 4). Inductive thematic analysis was then used to group the large number of documented barriers to and enablers of the implementation process^(71,72)^.

**Table 4.** Qualitative analysis of the barriers to and enablers of effective regulatory governance processes

Regulatory Governance measure	Enablers	Barriers
**Implementation**	**Regulatory substantive content** • Clear and consistent policy ^(50)^ • Local community involvement ^(46)^ • Feedback mechanism ^(51)^ • Establish metrics for measurement, such as data and timelines ^(14,51,53)^ • Mandatory policy that ‘levels the playing field’ ^(61,68)^ • Enforcement ^(55)^ • Healthy food clauses embedded in contract ^(13)^ • Contract length and cessation timing create opportunity for change ^(56,63)^ **Retailer** • Changes requiring no cost to retailer ^(42,69)^ • Retailer nutrition knowledge and beliefs ^(63)^ • Changes unlikely to be rejected by customers ^(42,69)^ • Retailer engagement and support ^(12,43,44,51,63,69,70)^ • Retailer perception of opportunity or competitive advantage of healthy food ^(12,63,68)^ • Implementation resources to support retailers: experts ^(12,43,58,60,64)^; labelling materials ^(40,43,58)^; site visits ^(43)^; implementation and classification guides ^(43,58,69)^ • Examples of success elsewhere ^(64,68)^ • Guidelines drove supply changes ^(68)^ **Customer** • Retail outlet located in more affluent areas ^(42)^ • Parental support for healthy food changes ^(43)^ • Customer demand ^(56)^ **Operational** • Store infrastructure/layout ^(53)^ **FINANCIAL** • Financial incentive for (customer or private industry) participation ^(39,64)^ **Choice** • Maximising healthy choices whilst not removing unhealthy choices ^(41,62)^ • Removal of choice on menu so no direct competition ^(41)^ • Easy changes to choice architecture are readily accepted by retailers ^(42)^ **Relationship management** • Stakeholder champions ^(12,51,56)^ • Supplier relationships ^(58,70)^ • External partnerships to assist implementation ^(46,58,63)^ **Communication** • Broad communication strategy ^(12,41,51,56,58,65)^ • Public recognition of success ^(12)^ • In person meetings ^(66)^ **Leadership** • Senior leadership approval or expectation of implementation ^(12,46,51,56,58,64)^	**Regulatory substantive content** • Contract length (locked in or temporary nature) ^(13,56,63,66)^ • Policy exemptions ^(40,43)^ • Lack of flexibility in guidelines ^(68)^ • Changing guidelines creating confusion ^(68)^ • Lack of nationally consistent standards ^(64)^ • Voluntary policy measures ^(63,64,66,67)^ **Retailer** • Resistance to healthier products in less affluent areas ^(42)^ • Profit loss ^(65,68)^ • Retailer nutrition knowledge and beliefs/’personal choice’ ^(61,63,64)^ • Concern that smaller portions lead to lower profits ^(42)^ • Cultural differences on definition of healthy food ^(42)^ • Concern that taste of healthy food is unpalatable to consumer ^(42,61,64)^ • Unhealthy products often cheap and highly profitable ^(42,43,45)^ • Highly competitive market and fear of profit loss ^(42,43,57,61–64,70)^ • Lack of resources to implement ^(62–64,68)^ • Resistance to selling fresh fruit due to high wastage ^(68)^ **CUSTOMER** • Customer dissatisfaction ^(42)^ **Operational** • Lack of healthy product supply ^(42,60,63,64,66,70)^ • Franchises and chain stores – difficult to implement local changes ^(58,68)^ • Practical and operational issues with store layout ^(42,66,68)^ • Incentives (i.e. marketing dollars, branding, machines) written into contracts from vendors, making it difficult to make healthy changes ^(56)^ • COVID-19 disruptions ^(66)^ **Relationship management** • Lack of champions ^(56)^ • Staff feeling responsible for unpopular decisions ^(56)^ **Communication** • Poor communication with retailers or customers ^(66,68,69)^ **Leadership** • No leadership support ^(63)^
**Monitoring**	• Provision of feedback to contractors on vending planograms ^(43,58,65)^ • Audit monitoring and tailored feedback to retailers ^(43)^ • Identification of stores non-compliant with policy ^(50)^ • Access to sales/revenue data ^(44,53,60,70)^ • Person assigned to monitor compliance ^(13)^ • Ongoing conversations with staff, site visits and menu analyses by experts ^(58)^ • Expectation that retailers would pass a compliance test by given date ^(68)^ • Independent monitoring scheme established with defined monitoring dates ^(68)^ • Monitoring (audit) tool developed by others and given to team ^(60,69)^ • Training of student interns at university to conduct audits ^(66)^	• Specific nutrition standards and contract monitoring NOT included in contract ^(13)^ • Lack of detail on independent monitoring and evaluation, including poorly defined targets ^(14)^ • Missing sales and revenue data reported ^(70)^ • Lack of time and staff resources to conduct monitoring ^(56,70)^
**Review**	• Inclusion of additional items in policy revisions ^(43,65)^ • Sales data monitoring and retailer feedback identified unexpected policy consequences that could be amended in policy review ^(68)^	• Lack of independent evaluation ^(14)^
**Enforcement**	• Contractual obligations to provide healthier options ^(43,51,68)^ • Compliance measures built into contract or tender process ^(43)^ • Policy compliance procedure ^(43)^ • Financial incentive to participate/comply ^(39)^ • Education of stakeholders enhances enforcement ^(55)^ • Mandatory regulation ^(68)^	• Staff actions and words may not reflect written regulation ^(55)^ • Self-regulation itself at odds with enforcement ^(45)^

## Results

### Selection of sources of evidence

**Fig. 1:**
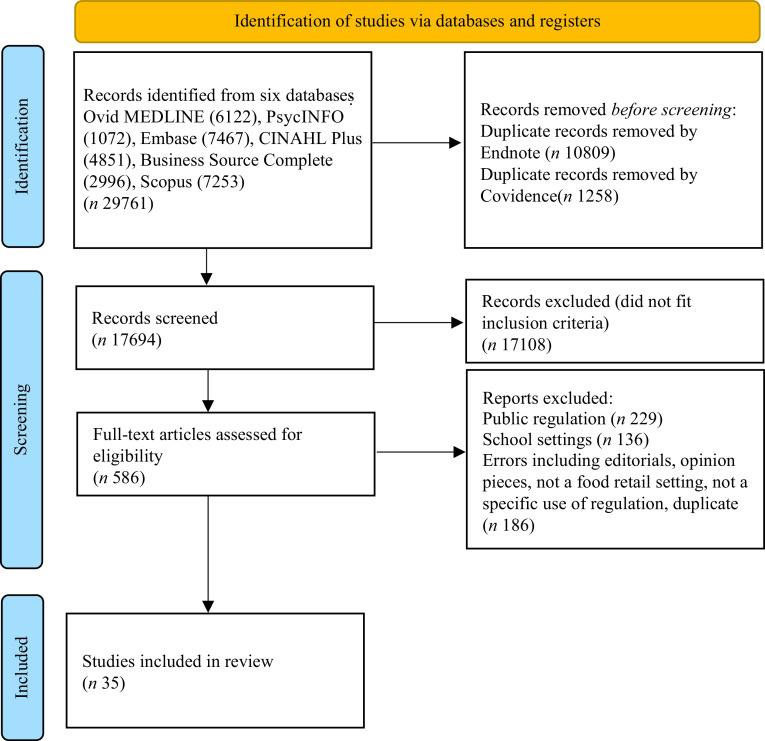
PRISMA

The final set of thirty-five articles were identified from 586 full-text articles assessed for eligibility from an initial screening of 17 694 articles. Reasons for exclusion of full-text articles are reported in our PRISMA flow diagram in Fig. 1.

### Characteristics of sources of evidence

The thirty-five articles identified were published between 2011 and 2023, with twenty-seven articles (77 %) published from 2015 onwards. Article characteristics are described in Tables 1 and 3.

### Results of individual sources of evidence and synthesis of results

#### Types of private regulatory measures used

The thirty-five articles identified reported on twenty-six unique private regulatory initiatives (hereafter ‘initiatives’) used to create healthier food retail environments, as some articles evaluated the same initiative but from a different perspective. Five articles reported on the Canadian Alberta Nutrition Guidelines for Children and Youth in Recreation Centres^(56,62,64,73,74)^. One of the articles that reported these Guidelines also reported on similar guidelines in Nova Scotia and British Columbia^(56)^. Four articles^(57,75,76)^ reported on United Kingdom (UK) voluntary supermarket checkout food policies, and three articles^(59,77,78)^ reported on the United States (US) National Restaurant Association’s Kids LiveWell program in the regional restaurant chain, Silver Diner. The remaining twenty-three articles reported on initiatives used in hospital and health service food retail outlets (*n* 6)^(12,43,52,58,68,79)^, vending machines (*n* 4)^(14,44,65,70)^, fast food outlets (*n* 3)^(47,80,81)^, supermarkets (*n* 3)^(15,39,54)^, independently owned food retail outlets (*n* 2)^(42,45)^, remote and regional community stores (*n* 2)^(46,53)^, universities (*n* 2)^(52,66)^ and sports and aquatic centres (*n* 1)^(60)^. Table 1 provides a summary of the characteristics of the included studies.

Of the twenty-six initiatives, ten were implemented in the USA^(45,47,51,52,58,65,66,70,78,81)^, seven in Australia^(12,43,44,46,53,60,79)^, five in the UK^(42,54,68,76,80)^, two in Canada^(13,56,62)^, one in Germany^(14)^ and one in South Africa^(39)^.

Sixteen (62 %) initiatives were voluntary^(13,14,39,42,45,47,53,58,60,64,66,75,78–81)^, six (23 %) mandatory^(46,51,52,65,68,70)^, two (7·5 %) used both voluntary and mandatory approaches^(12,43)^ and two initiatives (7·5 %) did not describe the status or provide enough information to determine the status (Table 3)^(44,54)^.

Of the twenty-six initiatives, forty (54 %) were developed and implemented by the organisation and twelve (46 %) were created based on healthy food retail frameworks or programs developed by governments, with implementation occurring at the organisational level via some form of private regulation.

#### Mozaffarian’s classification of policy interventions: domain and mechanism^(38)^

##### Domain

As noted in Table 1, of the twenty-six initiatives described, fourteen initiatives operated within one of the domains^(14,39,47,50,52–54,57–59,66,68,70,80,81)^ and twelve operated across multiple domains^(12,42–45,47,54,60,62,65,78,79)^. Thirteen operated in the domain of point of purchase information;^(12,42–45,47,60,61,65,67–69,78)^ sixteen took the form of food quality standards;^(12,14,33,42–44,47,52,54,56,58,60,61,65,66,69,70)^ six were in built environment changes;^(13,46,51,54,55,60)^ three were in population education^(39,53,65)^ and three were in the fiscal policy domain^(39,53,65)^.

##### Mechanism

Of the twenty-six initiatives, thirteen used one mechanism and thirteen used multiple mechanisms. Within the initiatives described: twenty-four targeted altering food availability or accessibility,^(12–14,39,41–44,46,47,51–56,58,60,61,66,68–70)^ nine targeted altering consumer preference or choice^(12,14,41,45,53,65,67–69)^ and ten targeted altering food formulation^(12,14,41–43,47,52,56,60,66)^.

#### Reporting of and responsibility for regulatory governance processes

In terms of the regulatory governance processes established by the initiatives, 34 articles reported on some aspect of implementation, 15 (43 %) articles reported a form of monitoring,^(40,43,44,51,52,54,56,58,60,64–66,68–70)^ two (11 %) articles reported on enforcement^(44,68)^ and six (17 %) articles reported on a review process^(39,41,43,52,66,68)^. Table 3 also describes the entity that had responsibility for the regulatory governance processes.

Two articles reported on implementation, monitoring, enforcement and review^(66,68)^. The article by Stead and colleagues, described the Healthcare Retail Standard (HRS), a regulatory scheme developed by the Scottish Government that applied to all food retail outlets in the Scottish National Health Service and aimed to increase healthy food options and limit the promotion of unhealthy food^(68)^. The HRS was a mandatory inclusion in any contract negotiated with a commercial retail outlet, which provided a process for enforcement, although the specific details of how the enforcement took place were not described. Non-commercial (National Health Service run) food retail outlets were also required to comply with the HRS, but this was not incorporated into their contracts, so no enforcement process was apparent. Monitoring of the HRS was managed by an external partner, the Scottish Grocer’s Federation, which is the trade association for the retail convenience sector in Scotland. The Scottish Grocer’s Federation conducted initial inspections and provided guidance to retailers on how to meet the HRS. It conducted biennial quality assurance inspections thereafter. The authors noted two examples of the HRS being reviewed and then modified: (1) the inclusion of lower fat baked potato crisps/chips in meal deals after the observation of an increase in full fat crisp/chip sales and (2) a revision allowing packaged snack items with the price marked prominently on their packaging, which were initially banned, after feedback from retailers that no alternative could be sourced. Whether the review process was regular, or reactive, was not described.

#### Barriers and enablers to effective regulatory governance processes

Table 4 lists the barriers to and enablers of effective regulatory governance processes, as described by the authors of the included studies. Barriers to and enablers of implementation were frequently identified in the literature but the barriers to and enablers of monitoring, review and enforcement were reported less often. The use of voluntary private regulatory measures was noted in some articles to be a barrier to both implementation^(63,64,67)^ and enforcement^(45)^. Studies reported a perception from retailers and managers that mandatory policies enabled implementation because they ‘levelled the playing field’^(61,68)^. Bogart and colleague’s article evaluating the American Beverage Association’s voluntary Better Calories initiative also noted concern within the public health community regarding compliance (and therefore effectiveness) of voluntary industry self-regulation given that industry’s primary aim is beverage sales (including unhealthy options)^(45,82,83)^.

Nine dominant themes emerged as either barriers to or enablers of implementation, including the regulatory substantive content, including the specific goals, terms, definitions, and conditions included in the regulation^(28)^; retailer issues, customer issues and operational issues – factors of concern related to retailers or customers, or practical/logistical issues related to operating a food retail outlet; financial issues related to financial cost/profit/loss associated with implementing the initiative; communication issues related to stakeholders, retailers and consumers being informed of initiatives; choice issues related to the perception of ‘free choice’ by consumers in selecting products; relationship management related to relationships between individuals and/or organisations within and/or outside their organisation such as internal stakeholders or food and drink supplier relationships and leadership – including organisational leadership and support.

The substantive content of a contract was identified as both an enabler and a barrier to implementation. Where a contract was due for renewal, this created an opportunity for change to occur, however, where an existing contract still had a significant time before renewal, this created a barrier to change^(56,66)^. One article noted that the very nature of contracts or leases created a defined period of time that may be too short for effectiveness to be demonstrated^(13)^.

Enablers to monitoring included audit processes, provision of expert feedback to vending contractors on compliance with policy, monitoring of sales data to determine policy impact and an expectation of compliance by a defined date. Barriers included lack of time and staff resources to conduct monitoring, poorly defined targets and specific nutrition standards being left out of contractual obligations.

The enablers of a review process included proper monitoring that enabled the unintended consequences of the regulatory measure to be identified and modified. In this way, the monitoring data fed into the review process so that modifications could be made. One article noted that there was a lack of independent evaluation^(14)^, but otherwise the articles did not comment on the absence of any review or evaluation processes.

The enablers of enforcement were the inclusion of obligations and enforcement measures in contractual arrangements, the education of stakeholders regarding the policy and the presence of a specific policy compliance procedure.

## Discussion

This study identified a range of private regulatory measures that aimed to create a healthier food retail environment. Our review found that private regulation was used under the auspices of programmes, standards, schemes, interventions, initiatives, policies, pledges charter, strategies, guidelines and contracts. The majority of initiatives described were voluntary despite recognition of the limitations of this format, particularly where commercial profit motives may be in conflict with the objectives of the initiative.

In the articles identified in this review, priority was given to reporting on implementation with less attention paid to other regulatory governance processes such as monitoring, review and enforcement. Accordingly, it was unclear from these studies whether many of the private regulatory measures described had established these important regulatory governance processes. Given that many of these articles were not focussed on regulatory governance, we do not discount the possibility that these processes may have been in place, but not reported on. In a recent review of healthy food retail interventions, Gupta and colleagues noted that the majority of published reviews also focused on implementation, with fewer focusing on programme sustainability and scale up^(22)^.

To enable improvement of healthy food retail initiatives, there needs to be greater reporting in the literature on the processes of monitoring, review and enforcement, along with evaluations of the barriers to and enablers of these regulatory governance processes. As discussed in the introduction section, these regulatory governance processes are key to the effective implementation of regulation, and effective regulatory implementation is more likely to result in improvements to the healthiness of the food retail environment, which the regulations under review in this study hope to achieve. Such reporting will also help to identify best regulatory practice design measures that facilitate the creation and sustainment of healthy food retail environments. The literature would benefit from the use of a robust, standardised framework that examines the entire regulatory process so that a comprehensive evaluation of the use of private regulation in healthy food retail environments can be made.

The barriers to and enablers of implementation reported in our study largely reflect those identified in two recent systematic reviews of healthy food retail interventions^(22,84)^. Retailer nutrition knowledge and beliefs, retailer concern over consumer demand or acceptance of healthier foods, profitability concerns and poor communication are reported as barriers to implementation across all three studies^(22,84)^. Similar enablers reported by all three studies were ease of intervention/implementation, no cost or profitable for retailer, consumer acceptance of changes, strong relationships/partnerships with all stakeholders and clear communication^(22,84)^. These barriers and enablers focus on the factors influencing implementation rather than the effectiveness of the implementation strategies themselves and/or the implementation strategies needed to bring about ongoing change. Our review brings attention to the need for researchers to go beyond reporting implementation and provide critical examination of the regulatory governance processes which in turn are important for effective implementation of healthy food retail initiatives^(22,84)^.

In an age of ‘big data’, we note that data, and access to it, was mentioned in only four articles as an enabler to monitoring^(44,53,60,70)^. Contractual obligations to electronically submit sales and nutrition data were noted as an enabler in the article by Wickramasekaran and colleagues evaluating a County-based healthy vending policy^(70)^. However, they also noted that data were missing for some months, indicating that despite contractual obligations, sales data can still be difficult to access and/or problematic for monitoring purposes^(70)^. Conversely, lack of detail or lack of data were identified as barriers to monitoring^(14,70)^. While the article by Stead and colleagues was the only one to include details of implementation, monitoring, review and enforcement, it did not detail the specific monitoring processes employed. The authors noted that future research could focus on retailer financial viability, and that longer-term monitoring is required for this purpose. This suggests that sales data were perhaps not monitored in their study and/or they were not privy to data on profit or other business metrics. Point of sale data is a rich source of information for monitoring the outcomes of regulation in the food retail environment, including profit and/or loss^(6,85)^. It is also worth noting that the monitoring conducted in the Stead article was managed by an external partner, the Scottish Grocer’s Federation, which is the trade association for the retail convenience sector in Scotland. Whilst independent monitoring is seen as best practice, in this example, the monitoring is independent of the retailer itself, but conducted by an industry trade association which may introduce a conflict of interest^(29)^. This also points to the importance of private regulation being accompanied by transparency and accountability processes and for further research evaluating the presence and operation of these processes.

In Australia, as in many industrialised economies who have pursued a ‘deregulation’ agenda, there has been little government appetite to pursue public regulation to create a healthier food retail environment^(86)^. This has created an opportunity for private regulation to fill the gap and diffuse throughout society, as various entities seek to create healthier food retail environments^(87)^. This diffusion of regulation away from government comes with risks and opportunities which need careful attention to enable equitable health outcomes^(88)^. In democratic societies governments have responsibilities to their citizens in a way that profit-driven companies do not, thereby enabling checks and balances on governments that are not otherwise applied to companies^(88)^. Commercial actors can act in ways beneficial to health; however, the literature notes the negative impact that powerful industries, such as the ultra-processed food industry, can have on health^(88,89)^.

Our finding that most of the articles were published relatively recently could reflect either (a) an increase in the use of private regulatory measures or (b) an increasing academic interest in reporting regulatory approaches to health-enabling food retail. This research may provide support to private actors involved in, or interested in implementing private regulatory measures, and empower them to include effective quality processes for monitoring, review and enforcement when drafting measures designed to create healthy food retail environments.

### Limitations

Due to their nature as agreements between private parties (and therefore often commercially sensitive and treated as confidential), there may be examples of private regulation being used to create healthy food retail environments that have not been subject to academic investigation and are therefore not captured by our search. However, this does not weaken the key finding that reporting on regulatory governance, specifically monitoring, review and enforcement processes, appears to be overlooked.

The large number of articles identified in the searches created a significant burden of articles to screen. JD and MF both have experience in the field of healthy food retail environments, and therefore, it was agreed that if we could decrease our inter-observer variability to < 5 % then JD could continue the screening alone (inter-observer variability reduced to 1·9 %). To minimise reviewer bias, two reviewers should screen all articles; however, the decision to review the articles by one researcher was made to ensure timely completion of the research.

### Conclusions

To be effective, private regulatory measures must be accompanied by effective processes for implementation, monitoring, review and enforcement^(27,28)^. Our research demonstrates that there is inadequate reporting in the peer reviewed literature on the processes for monitoring, review and enforcement, making it difficult to evaluate the presence or effectiveness of the regulatory processes established by each initiative. Strengthening reporting on the governance processes beyond implementation will improve the evidence base for forms of private regulation that aim to create a healthier food retail environment and enable the identification of design features that are more likely to lead to the creation of sustained healthier food retail environments.

## Supporting information

Dancey et al. supplementary material Dancey et al. supplementary material

## Supplementary material

For supplementary material accompanying this paper visit https://doi.org/10.1017/S136898002400065X.
