# Supplementary material for: The use of private regulatory measures to create healthy food retail environments: a scoping review
Source: Public Health Nutr. 2024 Mar 11;27(1):e88. doi: 10.1017/S136898002400065X (PMC11010160; doi:10.1017/S136898002400065X)
Supplement: Dancey et al. supplementary material [file S136898002400065Xsup001.docx]

# S1: A detailed search for Ovid Medline database

**POPULATION (FOOD RETAIL)**

1. fast food/
2. (food adj (outlet* or environment* or setting* or market* or court* or retail*)).ti,ab.
3. (store or stores).ti,ab.
4. takeaway.ti,ab.
5. coffee shop*.ti,ab.
6. cafe*.ti,ab.
7. quick service restaurant*.ti,ab.
8. kiosk*.ti,ab.
9. restaurant*.ti,ab.
10. cafeteria*.ti,ab.
11. canteen*.ti,ab.
12. tuckshop*.ti,ab.
13. (supermarket or hypermarket or superstore).ti,ab.

**INTERVENTION (REGULATION)**

1. tax*.ti,ab.
2. scheme*.ti,ab.
3. legislat*.ti,ab.
4. regulat*.ti,ab.
5. subsidy.ti,ab.
6. subsidi*.ti,ab.
7. agreement*.ti,ab.
8. lease*.ti,ab.
9. licenc*.ti,ab.
10. licens*.ti,ab.
11. contract*.ti,ab.
12. legal*.ti,ab.
13. clause*.ti,ab.
14. landlord*.ti,ab.
15. policy.ti,ab.
16. policies*.ti,ab.
17. ordinance*.ti,ab.
18. act.ti,ab.
19. acts.ti,ab.
20. ban.ti,ab.
21. bans.ti,ab.
22. law.ti,ab.
23. laws.ti,ab.
24. ((mandatory or voluntary or food or nutrition) adj (recommendation or guideline or standard)).ti,ab.

**OUTCOME (NUTRITION)**

1. beverage*.ti,ab.
2. obesity.ti,ab.
3. discretionary food*.ti,ab.
4. ultra-processed food*.ti,ab.
5. nutritio*.ti,ab.
6. nutrient*.ti,ab.
7. diet*.ti,ab.
8. sodium.ti,ab.
9. salt.ti,ab.
10. (fat or fats).ti,ab.
11. sugar*.ti,ab.
12. calorie*.ti,ab.
13. kilojoule*.ti,ab.
14. portion size*.ti,ab.
15. or/1-13
16. or/14-37
17. or/38-51
18. 52 and 53 and 54
19. limit 55 to english language
20. limit 56 to yr="2000 -Current"
